# Inflammasomes in rheumatoid arthritis: a pilot study

**DOI:** 10.1186/s41927-023-00353-8

**Published:** 2023-10-30

**Authors:** Qi Jiang, Xin Wang, Xiuping Xu, Liangfeng Hu, Guozhong Zhou, Rui Liu, Guocan Yang, Dawei Cui

**Affiliations:** 1 Department of Blood Transfusion, Shaoxing People’s Hospital, Shaoxing, 312000 China; 2 Department of Rheumatology and Immunology, Shaoxing People’s Hospital, Shaoxing, 312000 China; 3 Department of Clinical Laboratory Center, Shaoxing People’s Hospital, Shaoxing, 312000 China; 4 Department of Rheumatology, Suzhou TCM Hospital Affiliated to Nanjing University of Chinese Medicine, Suzhou, 215009 China; 5 Department of Blood Transfusion, the First Affiliated Hospital, Zhejiang University School of Medicine, Hangzhou, 310003 China

**Keywords:** Inflammasome, Rheumatoid arthritis, *NLRP3*, *NLRC4*, *AIM2*

## Abstract

**Background:**

The inflammasome plays an important role in rheumatoid arthritis (RA), which has rarely been systematically reported. The aim of this study was to understand whether the levels of inflammasomes were related to the severity of RA disease, which might provide a stronger theoretical basis for RA treatment.

**Methods:**

The *mRNA* expression levels of some inflammasomes and associated molecules, including *IL-1beta and IL-18*, in peripheral blood mononuclear cells (PBMCs) from 30 RA patients (n = 30) and 16 healthy control (HC) individuals were determined by quantitative real-time polymerase chain reaction (qRT‒PCR), and the levels of plasma IL-1beta and IL-18 were also measured by enzyme-linked immunosorbent assay (ELISA). Moreover, the clinical characteristics and laboratory results of the patients were collected and analyzed in this study.

**Results:**

The relative *mRNA* expression levels of *NLRP3, NLRC4, AIM2, caspase-1*, and *IL-1beta* were significantly higher and those of *NLRP1, NLRP2 and NLRC5* were notably lower in the HC group than in the RA group. Moreover, the plasma IL-1beta and IL-18 levels were markedly increased in the RA group. Additionally, the *mRNA* level of *AIM2* was negatively correlated with disease activity score 28 (DAS28) by stepwise linear regression analysis. erythrocyte sedimentation rate (ESR) was positively correlated with DAS28 by multiple linear regression analysis in the RA group.

**Conclusions:**

These findings imply the critical role of *NLRP3, NLRC4, AIM2, caspase-1* and plasma IL-1beta and IL-18 in the pathogenesis of RA patients, which provides potential targets for the treatment of RA.

**Supplementary Information:**

The online version contains supplementary material available at 10.1186/s41927-023-00353-8.

## Background

Rheumatoid arthritis (RA) is a systemic autoimmune disease characterized by chronic erosive arthritis. The pathogenesis is marked by persistent, chronic inflammation of the synovial membrane. This leads to synovitis and damage to bone and joints, which ultimately causes joint deformity, loss of function, and even disability [1, 2]. The pathogenesis of RA remains unclear, although inflammasomes are considered to be closely involved in RA pathogenesis. Inflammasomes are an important component of the immune system. They identify pathogen-associated molecular patterns (PAMPs), damage-associated molecular patterns (DAMPs), or homeostasis-altering molecular processes (HAMPs) on pathogens through pattern recognition receptors (PRRs), recognizing and eliminating exogenous pathogenic microorganisms or endogenous molecules released by the host itself [3, 4]. The canonical inflammasomes are composed of a sensor molecule, an adapter protein (apoptosis-associated speck-like protein containing a caspase recruitment domain, ASC), and an effector molecule [5]. The sensors include members of the NLR family, the absent in melanoma 2 (AIM2)-like receptor (ALR) family and pyrin, specifically NOD-like receptor protein 1 (NLRP1), NLRP2, NLRP3, NOD-like receptor family apoptosis inhibitory protein (NAIP), NLR family CARD domain-containing 4 (NLRC4), NLRC5, NLRP6, NLRP7, NLRP9, NLRP12, AIM2, human interferon (IFN) γ-inducible protein (IFI) 16 and pyrin [6, 7]. Caspases include human caspases-1/4/5 and mouse caspases-1/11 and active inflammatory caspases that cleave pro-IL-1beta and pro-IL-18 to produce active IL-1beta and IL-18. Thus, they exert proinflammatory effects and promote the cleavage of the pore-forming protein gasdermin D (GSDMD) to induce pyroptosis [5, 8].

Inflammasomes are expressed in immune cells, such as T cells, monocytes, and macrophages, and nonimmune cells, such as epithelial cells and myofibroblasts [5]. An appropriate inflammatory response is beneficial for the body. However, it will trigger excessive tissue damage and lead to disease when it is uncontrolled. While there are numerous reports on inflammasomes in RA, no systematic studies have been reported.

In this study, we assessed the *mRNA* levels of *NLRP1, NLRP2, NLRP3, NLRC4, NLRC5, NLRP12, AIM2, CARD8, IFI16, Pyrin, NAIP, caspase-1, caspase-4, caspase-5, IL-1beta, and IL-18* in PBMCs from RA patients and HC individuals and conducted a pilot investigation of common inflammasomes and associated molecules to provide a stronger theoretical basis for RA treatment.

## Materials and methods

### Objective

This study aimed to perform a pilot study on the expression levels of inflammasomes and associated molecules to provide a stronger theoretical value for RA treatment.

### Study design and participants

A total of 30 patients with RA were enrolled in this study; these patients included 10 males and 20 females. The average age of these patients was 55 years (range: 31 to 75 years). RA patients who fulfilled the revised American College of Rheumatology (ACR) 2010 criteria for RA were consecutively enrolled in the Shaoxing People’s Hospital between October 2021 and March 2022 [9]. RA patients with other inflammatory or autoimmune diseases, cancers, mental disorders or hormonal diseases and pregnant and lactating women were excluded from this study. Information on disease activity score 28 (DAS28), rheumatoid factor (RF), anti-cyclic citrullinated peptide antibodies (anti-CCP), erythrocyte sedimentation rate (ESR), C-reactive protein (CRP), white blood cell (WBC) count, neutrophil count, monocyte count, lymphocyte count and other data were collected. A total of 16 healthy control individuals (HC individuals) matched for sex and age were also enrolled. This group included 5 males and 11 females of 40 to 72 years of age who had no autoimmune or inflammatory diseases. The characteristics of these enrolled patients are summarized in Table 1.

**Table 1:** Clinical details of the RA and HC groups

	RA (n = 30)	HC (n = 16)
**Demographics**		
Sex, male/female	10/20	5/11
Age (range)	55 (31–75)	57 (40–72)
BMI (kg/m^2^)	22.4 ± 4.1	
Smoking (Yes/No)	8/22	
Alcohol drinking (Yes/No)	10/20	
**Clinical characteristic**		
Disease duration, years (range)	6.37(0.3–16)	
DAS28-CRP	3.66 ± 1.79	
RF, IU/ml	255.20 ± 237.70	
Anti-CCP, RU/ml	366.90 ± 476.27	
ESR, mm/h	36.60 ± 27.52	
CRP, mg/l	16.91 ± 22.07	
plasma IL-1beta, pg/ml	6.62 ± 1.65^***^	4.93 ± 0.98
plasma IL-18, pg/ml	759.79 ± 661.36^**^	307.89 ± 230.42
WBC, 10^9^/l	5.96 ± 1.87	5.38 ± 1.25
N, 10^9^/l	3.87 ± 1.64	2.97 ± 1.01
M, 10^9^/l	0.43 ± 0.17	0.39 ± 0.09
L, 10^9^/l	1.78 ± 1.16^*^	1.86 ± 0.37
**Treatments**		
Glucocorticoids, n (%)	12 (40%)	
Methotrexate, n (%)	18 (60%)	
Biologic agents, n (%)	8 (27%)	
JAK inhibitors, n (%)	5 (17%)	

Note: DAS28-CRP, disease activity score for 28 joints based on C-reactive protein; RF, rheumatoid factor; BMI, body mass index; anti-CCP, anti-cyclic citrullinated peptide antibody; ESR, erythrocyte sedimentation rate; IL-1beta, interleukin-1beta; WBC, white blood cell count; N, neutrophil count; M, monocyte count; L, lymphocyte count. * P < 0.05, ** P < 0.01, *** P < 0.001

### Human PBMC separation and total RNA extraction

Fresh peripheral blood samples were collected into EDTA-K_2_-containing tubes and centrifuged at 800 × g for 5 min. Then, the plasma was collected into the tubes and stored at -80 °C. The remaining blood components were diluted with an equal volume of phosphate buffered saline (PBS), and then PBMCs were collected by gradient centrifugation according to the Lympholyte®-H (CEDARLANE, Netherlands) manufacturer’s protocols. The total RNA in PBMCs from each case was extracted using an RNA Extraction Kit (Vazyme Biotech, Nanjing, China) as quickly as possible according to the protocol. The integrity and concentration of the RNA were assessed by a NanoDrop 2000 (Thermo Scientific, USA) spectrophotometer.

### qRT‒PCR analysis

Quantitative real-time polymerase chain reaction (qRT‒PCR) was carried out with the PrimeScript™ RT kit (Vazyme Biotech, Nanjing, China) and SYBR Premix Ex Taq™ II (Vazyme Biotech, Nanjing, China). The housekeeping gene encoding GAPDH was used as an internal control, and the primers are listed in Table 2. The relative expression levels of the target genes were analyzed using the 2^−ΔΔCt^ method.

**Table 2:** The amplification primer sequences

Gene name	GeneBank accession no.	Primer sequence (5’-3’)
*NLRP1*	NM_033004	F-ATTGAGGGCAGGCAGCACAGAT
		R-CTCCTTCAGGTTTCTGGTGACC
*NLRP2*	NM_017852	F-CATTCTGCGTCAAGCACTGTCG
		R-CCGTCCAGAAAGGAAGCATGTG
*NLRP3*	NM_004895	F-GGACTGAAGCACCTGTTGTGCA
		R-TCCTGAGTCTCCCAAGGCATTC
*NLRC4*	NM_021209	F-AGGTCCCACAACTCGTCAAGCT
		R-TGCTCACACGATTTCCCGCCAA
*NLRC5*	NM_032206	F-AGTGGCTCTTCCGCTTGGACAT
		R-CGGAACCCTAAGAACTTGGCTG
*NLRP12*	NM_144687	F-CAGGCATGATGCTGCTTTGCGA
		R-AGCACAGAAGCCATCTCCTGAC
*AIM2*	NM_004833	F-GCTGCACCAAAAGTCTCTCCTC
		R-CTGCTTGCCTTCTTGGGTCTCA
*CARD8*	NM_014959	F-GTGTGGGATACTGAGGTGAAGC
		R-TGTCCTGGAGATCATCGAGCAC
*IFI16*	NM_005531	F-GATGCCTCCATCAACACCAAGC
		R-CTGTTGCGTTCAGCACCATCAC
*Pyrin*	NM_000243	F-GCTGCTCTTCTGTGAGGATCAC
		R-CTCAGCTTCTTCAGATGCTCCAG
*NAIP*	NM_004536	F-CCGAACAGGAACTGCTTCTCAC
		R-CCACAGACAGTTCTTTCAGGCAC
*Caspase-1*	NM_033292	F-GCTGAGGTTGACATCACAGGCA
		R-TGCTGTCAGAGGTCTTGTGCTC
*Caspase-4*	NM_001225	F-GGGATGAAGGAGCTACTTGAGG
		R-CCAAGAATGTGCTGTCAGAGGAC
*Caspase-5*	NM_004347	F-ACAACCGCAACTGCCTCAGTCT
		R-GAATCTGCCTCCAGGTTCTCAG
*IL1beta*	NM_000576	F-CCACAGACCTTCCAGGAGAATG
		R-GTGCAGTTCAGTGATCGTACAGG
*IL-18*	NM_001562	F-GATAGCCAGCCTAGAGGTATGG
		R-CCTTGATGTTATCAGGAGGATTCA
*GAPDH*	NM_002046	F-GTCTCCTCTGACTTCAACAGCG
		R-ACCACCCTGTTGCTGTAGCCAA

Note: NLRP1, NOD-like receptor protein 1; AIM2, absent in melanoma 2; IFI16, human interferon (IFN) g-inducible protein 16; NAIP, NOD-like receptor family apoptosis inhibitory protein

### Enzyme-linked immunosorbent assay (ELISA)

The plasma concentrations of IL-1beta and IL-18 were determined by enzyme-linked immunosorbent assay (ELISA) according to the protocol of the kit (Boster Biological, Wuhan, China). The detection wavelength was 450 nm, and experiments were conducted in triplicate with consistent results.

### Statistical analysis

Statistical analysis and graphic presentation were performed by SPSS version 25.0 and GraphPad Prism 9.0. Unpaired Student’s t test or one-way ANOVA was used when the data passed the normality test; otherwise, the nonparametric Mann–Whitney test or the Kruskal‒Wallis test was used. The correlation analysis was performed by multiple linear regression analysis and stepwise linear regression analysis, excluding the interference of confounding factors. Two-tailed P < 0.05 was considered to be statistically significant.

## Results

### Characteristics of study subjects

Thirty patients with RA and sixteen healthy adults were enrolled in this study. There was no sex or age differences between the RA group and the HC group. There was no significant difference in the counts of WBCs, monocytes and neutrophils between the two groups, but the number of lymphocytes in the RA group was higher than that in the HC group (P = 0.04).

### The *mRNA* expression levels in the RA and HC groups

**Fig. 1:**
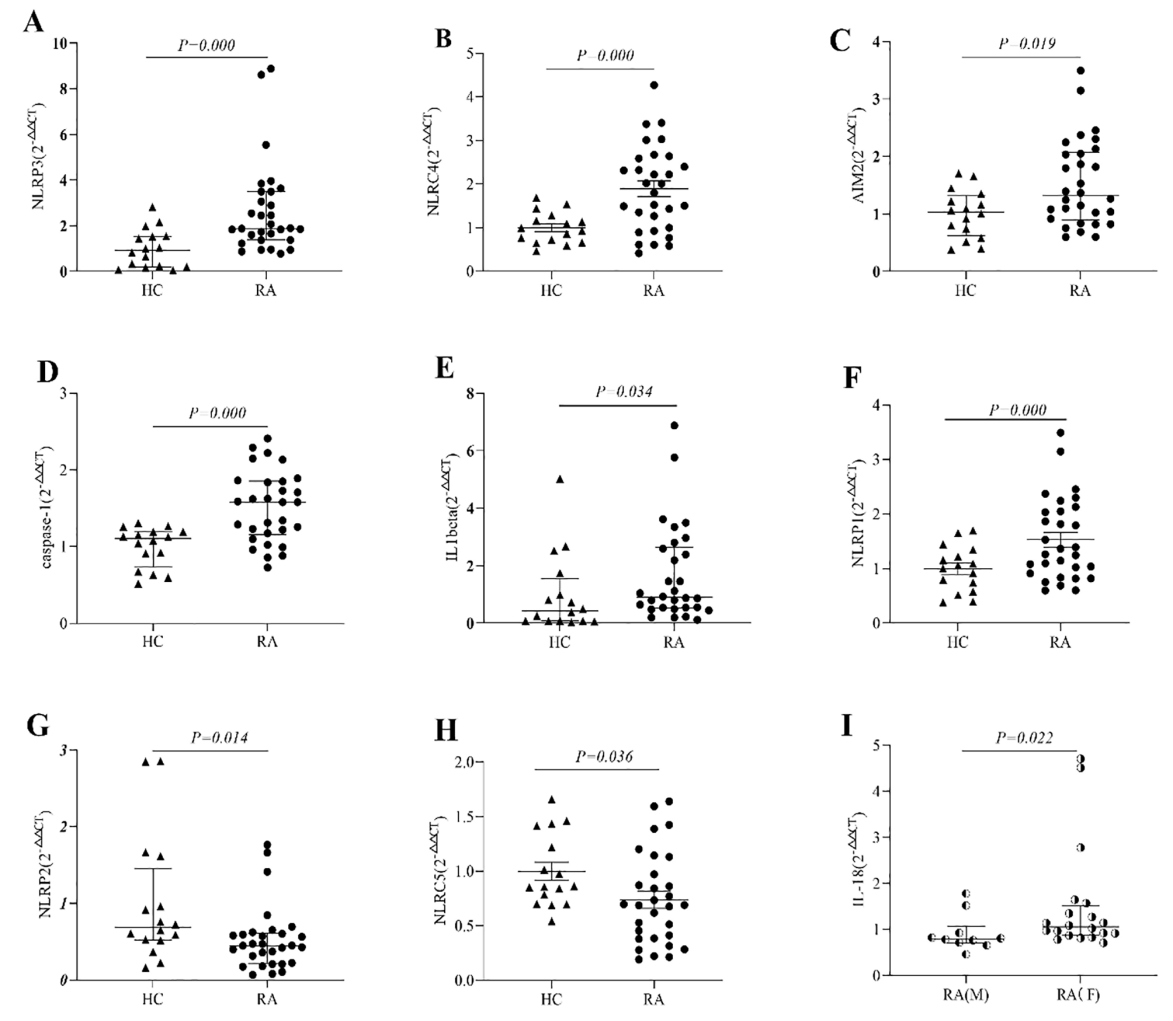
**Differential expression levels of inflammasomes and associated molecules**. (**A**) The *mRNA* level of *NLRP3*. (**B**) The *mRNA* level of *NLRC4*. (**C**) The *mRNA* level of *AIM2*. (**D**) The *mRNA* level of *caspase-1*. (**E**) The *mRNA* level of *IL-1beta*. (**F**) The *mRNA* level of *NLRP1.* (**G**) The *mRNA* level of *NLRP2*. (**H**) The *mRNA* level of *NLRC5*. (**I**) The *mRNA* level of *IL-18* in females with RA. RA, rheumatoid arthritis group; HC, healthy control group; M, male; F, female

The *mRNA* expression levels of *NLRP3, NLRC4, AIM2, caspase-1 and IL-1beta* in the RA group were markedly increased (Fig. 1A-E), but the levels of *NLRP1, NLRP2 and NLRC5* were notably decreased in comparison with those in the HC group (Fig. 1F-H). Moreover, there were no significant differences in *NLRP12, CARD8, IFI16, pyrin, NAIP, Caspase-4/5 and IL-18 mRNA* levels between the two groups (Supplementary material: Table S1). Although there was no significant difference in the relative expression levels of *IL-18 mRNA* between the two groups, the female group was markedly higher than the male group in RA patients (Fig. 1I).

### Inflammasomes and DAS28-CRP in RA patients

**Fig. 2:**
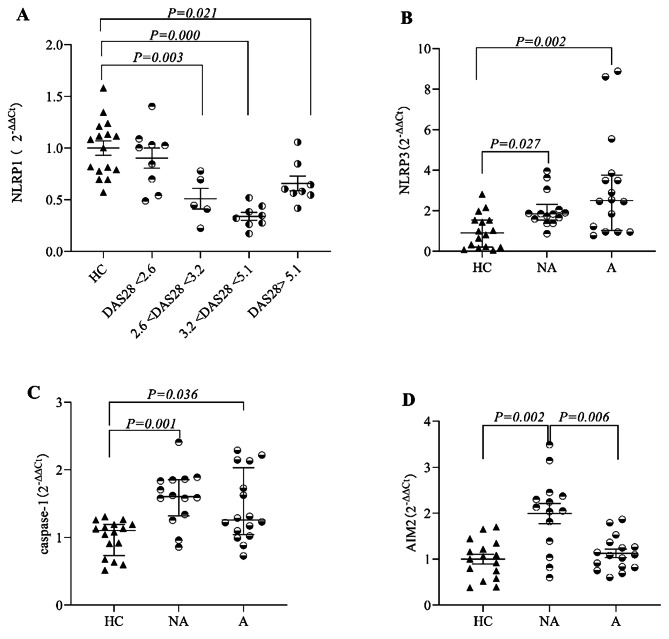
**Differences in the expression of inflammasomes in different groups**. (**A**) *NLRP1 mRNA* levels in the DAS28-CRP > 5.1 (high activity group), 3.2 < DAS28-CRP < 5.1 (moderate activity group), and 2.6 < DAS28-CRP < 3.2 (low activity group) groups were noticeably lower than those in the HC group, but there were no significant differences between the DAS28-CRP < 2.6 (remission group) and HC groups. (**B**) *NLRP3 mRNA* levels in the inactive and active groups were higher than those in the HC group. (**C**) *Caspase-1 mRNA* levels in the inactive and active groups were higher than those in the HC group. (**D**) *AIM2 mRNA* levels in the inactive group were higher than those in the HC group and the active group. IA, inactive group; A, active group

To assess whether these inflammasomes and related proteins differed at different disease activity levels, the RA group was divided into high activity (DAS28-CRP > 5.1), moderate activity (3.2 < DAS28-CRP < 5.1), low activity (2.6 < DAS28-CRP < 3.2), and remission (DAS28-CRP < 2.6) groups according to DAS28-CRP [9]. The *NLRP1 mRNA* levels in the RA with different activity groups (low, moderate and high activity) were noticeably different from those in the HC group, and there were no significant differences between the RA remission group and the HC group. The relative expression levels of *NLRP1 mRNA* were not markedly different among the different RA activity groups (low, moderate and high activity) (Fig. 2A). The comparison between the active (including moderate and high activity groups) and inactive (low activity and remission groups) RA groups indicated that the *mRNA* levels of *NLRP3 and caspase-1* in both the inactive and active groups were higher than those in the HC group, but there were no notable differences between the inactive and active groups (Fig. 2B, C). The levels of *AIM2 mRNA* in the inactive group were the highest and were significantly different from those in the HC group and the active group (Fig. 2D). The CRP and ESR levels in the high activity group were markedly higher than those in the low activity group and the remission group.

### Measurement of plasma IL-1beta and IL-18

**Fig. 3:**
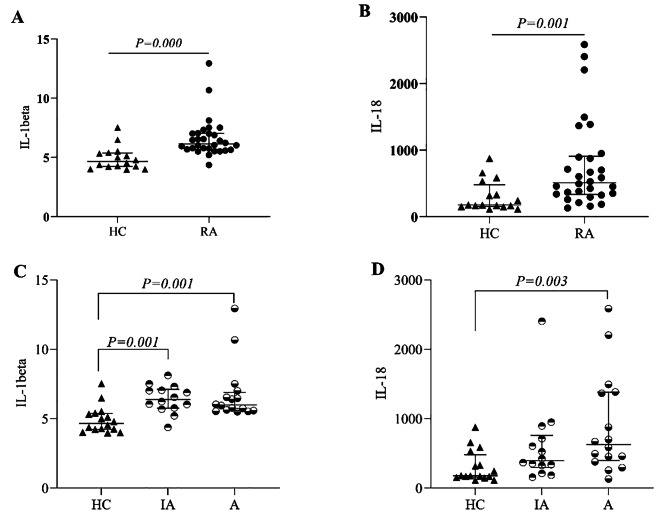
**Differential expression assay of plasma IL-1beta and IL-18**. (**A**), The plasma levels of IL-1beta in the RA group were noticeable higher than those in the HC group. (**B**) The plasma levels of IL-18 in the RA group were markedly higher than those in the HC group. (**C**) The plasma levels of IL-1beta in the inactive and active groups were higher than those in the HC group. (**D**) The plasma levels of IL-18 in the active group were higher than those in the HC group

To determine whether there were differences in the plasma IL-1beta and IL-18 levels between RA patients and HC groups, these levels were determined by ELISA. The plasma IL-1beta (6.62 ± 1.65, P = 0.000) and IL-18 (759.79 ± 661.36, P = 0.001) levels in the RA group were noticeably higher than those in the HC group (Fig. 3A, B). There were noticeable significant differences among the active, inactive, and HC groups (Fig. 3C), but no difference was observed between the active and inactive groups. The level of IL-18 was the highest in the active group, which was markedly different from that in the HC group (Fig. 3D).

### Correlation between *mRNA* expression levels and clinical features of RA patients

To explore the relationships among the *mRNA* expression levels of these inflammasomes, plasma IL-1beta, IL-18 levels and clinical characteristics of RA patients, multiple linear regression analysis was used to analyze the relative *mRNA* expression levels of *NLRP1, NLRP2, NLRP3, NLRC4, NLRC5, NLRP12, AIM2, CARD8, IFI16, Pyrin, NAIP, Caspase-1, Caspase-4, Caspase-5, IL-1beta, IL-18 mRNA* and plasma IL-1beta and IL-18 levels and the clinical characteristics of RF, anti-CCP, CRP and ESR. However, no significant difference was found.

To find out whether the inflammasome *mRNAs* above affect plasma IL-1β and IL-18 levels, the results of stepwise regression analysis showed that the *mRNA* expression of inflammasome *NLRP1* (β=-0.351, P = 0.017, 95% CI -3.068 to -0.323, R squared after adjustment 0.123) was negatively correlated with plasma IL-1β and that the *mRNA* expression of *IL-18* (β=-0.351, P = 0.017, 95% CI -3.068 to -0.323, R squared after adjustment 0.123) was positively correlated with plasma IL-18 levels.

To understand the correlation between the expression level of these inflammasomes and related molecules and disease activity, multiple linear regression analysis and stepwise linear regression analysis were conducted. The results showed that the *mRNA* level of *AIM2* was negatively correlated with DAS28 (β=-0.420, P = 0.021, 95% CI -1.832 to -0.162, R squared after adjustment 0.147). Additionally, to determine whether the laboratory findings were associated with DAS28, we compared the RF, anti-CCP, CRP, ESR, and neutrophil, monocyte and lymphocyte counts with DAS28 using multiple linear regression analysis. The results showed that ESR was positively correlated with DAS28 (β = 0.711, P = 0.01, 95% CI 0.013 to 0.083, R squared after adjustment 0.623).

## Discussion

It is well known that the inflammasome is an important part of the innate immune system. Inflammasome activation triggers a series of inflammatory cascade responses, leading to the secretion of various cytokines, including the production of IL-1beta and IL-18. This has implications for the pathogenesis of many diseases, such as RA, systemic lupus erythematosus (SLE) and influenza virus infection [10]. In view of the role of inflammasomes in RA disease, we investigated the relative *mRNA* expression levels of common inflammasomes, including *NLRP1, NLRP2, NLRP3, NLRC4, NLRC5, NLRP12, AIM2, CARD8, IFI16, Pyrin* and related molecules, including *NAIP, caspase-1, caspase-4, Caspase-5, IL-1beta and IL-18*, in human PBMCs of RA patients and HC individuals.

The results showed that the relative *mRNA* expression levels of *NLRP3, NLRC4, AIM2, caspase-1, and IL-1beta* in the RA group were noticeably higher than those in the HC group. However, the relative *mRNA* expression levels of *NLRP1, NLRP2, and NLRC5* were notably decreased in the RA group compared to those in the HC group. Interestingly, the *mRNA* levels of *IL-18* were significantly higher in the female group than in the male group in RA patients. Although the lymphocyte count in the RA group was higher than that in the HC group, the two groups were comparable because the present study compared relative expression levels in human PBMCs. PBMCs mainly contain lymphocytes (including T-lymphocytes, B-lymphocytes, NK cells, etc.) and monocytes. In this study, inflammasomes mainly in monocytes and T lymphocytes of RA patients were activated, causing a cascade of reactions that affected the plasma levels of IL-1beta and IL-18 [10]. Therefore, we examined the plasma levels of IL-1beta and IL-18 in the RA and HC groups and found that the plasma IL-1beta and IL-18 levels in the RA group were markedly higher than those in the HC group. To further explore whether plasma IL-1beta and IL-18 can reflect disease activity and inflammation, we correlated inflammasome *mRNA* levels and cytokines with DAS2, CRP, ESR, RF and anti-CCP. The results showed that the *mRNA* level of *AIM2* was negatively correlated with the DAS28 and that the ESR was positively correlated with the DAS28.

Wang et al. showed that *NLRP3 and IL-1beta mRNA* levels in human PBMCs were not significantly different between the RA patients and HC individuals, and *caspase-1 mRNA* levels were lower than controls. These results are different from the results of the present study, probably due to factors such as disease duration or drug use [11]. We divided *NLRP3, caspase-1 and IL-1beta mRNA* into five or three groups according to DAS28-CRP, and there was no noticeable difference between the groups (data not shown). These findings suggest that these *mRNAs* were not highly expressed due to increased disease activity. Some studies have shown that tofacitinib (TOF) attenuates NLRP3 activation and reduces serum IL-1beta production [12]. NLRP3 expression is increased in adjuvant arthritis (AA) and collagen-induced arthritis (CIA) animal models [13, 14]. Both IL-1beta and NLRP3 levels are increased in the PBMCs of RA patients [15, 16], suggesting that NLRP3 exacerbates the inflammatory response in RA. Moreover, inhibitors targeting NLRP3 have been applied to a variety of clinical diseases with good clinical efficacy [8]. Therefore, NLRP3 plays a pathogenic role in the disease process of RA.

Our study showed that AIM2 levels were significantly higher in the RA group than in the HC group, which was consistent with a previous report [17]. After we divided *AIM2* into three groups according to disease activity, the results showed a significant difference between the active, inactive and HC groups. The highest *AIM2* levels were found in the inactive group, which might explain why the relative *mRNA* levels of *AIM2* were negatively correlated with DAS28. Animal models of AIM2 deficiency exhibit milder inflammatory responses and pathological changes [18, 19]. Although the AIM2 levels in RA patients were lower than those in HC individuals, fibroblast-like synovial cell (FLS) proliferation was inhibited after AIM2 was silenced in FLSs from RA patients [20]. Although the results were inconsistent, AIM2 remains a target for RA treatment according to the current findings. Additionally, there are fewer studies on NLRC4 in RA, and only one study from Brazil supports the role of NLRC4 in RA [21]. Our results showed higher *NLRC4* levels in the RA group than in the HC group. In conclusion, both *AIM2 and NLRC4* may play pathogenic roles in the RA disease process.

Gene polymorphisms of *NLRP2* are associated with susceptibility to RA in the Chinese Han population [22]. There are no other reports on NLRP2 in RA, but the present study first found that the *NLRP2* levels in PBMCs of RA patients were significantly lower than those in HC individuals. However, its function still needs to be further studied. In addition, we found that the *mRNA* levels of *NLRP1* were also markedly lower in RA patients than in HC individuals, which is consistent with a previous report [11]. And *NLRP1 mRNA* level was negatively correlated with plasma IL-1beta. After the RA group was grouped by DAS28, there was no significant difference between these groups, indicating that *NLRP1* was not associated with disease activity. Different results on whether genetic polymorphisms of *NLRP1* are associated with RA have been reported, and some studies have also reported that inflammatory conditions in animal models can be attenuated by inhibiting the NLRP1 inflammasome [23–25]. In general, the role of NLRP1 in RA needs to be further explored with larger sample sizes that exclude the influence of other relevant factors. NLRC5 levels significantly increased in synovial tissues of AA rats and FLSs of RA patients, and inflammatory cytokines were significantly decreased after NLRC5 silencing [26–28].

Genetic polymorphisms of *CARD8* have been most studied for their association with RA susceptibility or anti-TNF therapy [21, 29]. Caspase-4/5 is a downstream protein of the noncanonical pathway of the inflammasome that is rarely reported in RA, and genetic polymorphisms of caspase-5 are associated with an increased risk of RA development [30]. Studies have shown that NLRP12 negatively regulates the phosphorylation of signal transducer and activator of transcription 3 (STAT3), and the inflammatory response is exacerbated in the NLRP12^−/−^ antigen-induced arthritis (AIA) mouse model [31, 32]. The roles of the inflammasomes mentioned above need to be further investigated.

The levels of plasma IL-1beta and IL-18 were significantly higher in RA patients than in HC individuals, and there was no significant difference between the groups when IL-1beta was divided into five or three groups. The use of leflunomide has been reported to decrease the serum level of IL-18 [33]. In this experiment, the study subjects were divided into a group using leflunomide, a group using hormones and a group using both drugs. The plasma level of IL-18 was analyzed between the three groups, and there was no significant difference in our study. Vasilev et al. reported that serum IL-18 levels were significantly reduced in females [34]. In this study, there was no significant difference in plasma IL-18 levels after grouping by sex, but *IL-18 mRNA* levels were notably lower in the female group than in the male group. Furthermore, *IL-18 mRNA* was correlated with plasma IL-18. The results of this study may also suggest the use of drugs that target IL-18 in the treatment of female patients with RA.

## Conclusions

Although common inflammasomes have been gradually reported in RA, there are no systematic studies reporting multiple inflammasomes simultaneously. Our study is a more comprehensive report of common inflammasomes and their associated proteins in RA. Future studies with larger sample sizes in newly diagnosed patients with RA or with a single drug alone are needed due to sample size limitations and drug use. The human body is complex. The pathogenesis of diseases is not controlled by a single cytokine and involves complex interactions between proinflammatory and anti-inflammatory factors. For example, caspase-1 is a downstream protein of many inflammasomes, so its level can be increased or decreased by a variety of factors. Because of this, inhibitors targeting caspase-1 have also become the focus of research. For example, VX-740 (pralnacasan) attenuates osteoarthritis injury, and VX-765 (belnacasan) inhibits cytokine secretion in RA models [35, 36]. Additional inhibitors include MCC950, which directly targets the NACHT domain of the NLRP3 inflammasome, tranilast (TR) [37, 38], anakinra, which is an antagonist against the IL-1 receptor (IL-1Ra), and canakinumab, which is an anti-IL-1β monoclonal antibody [39, 40]. The latest study shows that *NLRP3* gene polymorphisms elevated the susceptibility to RA disease, and have an impact on RF as well as anti-CCP titers in RA patients, and pyroptosis will also be a possible therapeutic target for RA [41, 42]. With the deepening of the research on the mechanisms of the inflammasome, inhibitors of the inflammasome itself or its related upstream and downstream proteins are gradually being applied.

RA is caused by a variety of factors, genetic or environmental, and its pathogenesis and therapeutic approaches are constantly evolving. Given the important role of inflammasomes in the disease process of RA, a deeper understanding of the mechanisms of action of each inflammasome is important for the development of RA treatment strategies. Overall, our findings indicate the important role of inflammasomes and associated molecules in the pathogenesis of RA patients and could contribute to the therapeutic strategy of RA.

### Electronic supplementary material

Below is the link to the electronic supplementary material.

Supplementary Material 1

## Data Availability

All datasets generated for this study are included in the manuscript and the Supplementary material.

## Abbreviations

PBMCs: Peripheral blood peripheral blood mononuclear cells
ELISA: Enzyme-Linked Immunosorbent Assay
PAMPs: Pathogen-associated molecular patterns
DAMPs: Damage-associated molecular patterns
HAMPs: Homeostasis-altering molecular processes
PRRs: Pattern recognition receptors
NLRP3: NOD-like receptor protein 3
NLRC4: NLR family CARD domain-containing 4
AIM2: Absent in melanoma 2
IFI16: Human interferon (IFN)g-inducible protein 16
NAIP: NOD-like receptor family apoptosis inhibitory protein
GSDMD: Pore-forming protein gasdermin D
CRP: C-reactive protein
ESR: Erythrocyte sedimentation rate
RF: Rheumatoid factor
anti-CCP: Anti-cyclic citrullinated peptide antibodies
DAS28: Disease activity score 28
