# Supplementary material for: Inflammasomes in rheumatoid arthritis: a pilot study
Source: BMC Rheumatol. 2023 Oct 30;7:39. doi: 10.1186/s41927-023-00353-8 (PMC10614352; doi:10.1186/s41927-023-00353-8)
Supplement: Supplementary file 1 — Supplementary Material 1 [file 41927_2023_353_MOESM1_ESM.docx]

**Table S1. No significant difference mRNA levels between RA and HC group.**

| Gene Name | P value |
| --- | --- |
| NLRP12 | 0.580 |
| CARD8 | 0.052 |
| IFI16 | 0.764 |
| Pyrin | 0.872 |
| NAIP | 0.987 |
| Caspase-4 | 0.119 |
| Caspase-5 | 0.170 |
| IL-18 | 0.782 |
